# Antithrombotic therapy and the risk of pocket hematoma after subcutaneous implantable cardioverter-defibrillator implantation

**DOI:** 10.1007/s10840-024-01973-x

**Published:** 2025-01-16

**Authors:** S. Pepplinkhuizen, N. Kors, J. A. de Veld, L. A. Dijkshoorn, N. R. Bijsterveld, A. de Weger, L. Smeding, A. A. M. Wilde, L. R. A. Olde Nordkamp, R. E. Knops

**Affiliations:** Heart Center, Department of Cardiology, Amsterdam Cardiovascular Sciences Heart Failure & Arrhythmias, Amsterdam UMC Location University of Amsterdam, Meibergdreef 9, 1105 AZ Amsterdam, the Netherlands

**Keywords:** Subcutaneous ICD, Implantable cardiac defibrillator, Complications, Pocket hematoma

## Abstract

**Background:**

Little data exists regarding the optimal antithrombotic strategy during S-ICD implantation to prevent pocket hematomas. This study explores the association between perioperative antithrombotic management and the occurrence of pocket hematoma following S-ICD implantation.

**Methods:**

All patients who underwent de novo S-ICD implantation between February 2009 and January 2023 at Amsterdam UMC were included. Data was collected retrospectively from electronic patient records. Clinically significant pocket hematomas were defined as an accumulation of blood at the pocket site within 30 days after implantation.

**Results:**

A total of 347 patients were included of which 224 (64.6%) patients used antithrombotic therapy pre-implantation. The median age at implantation was 50 years (IQR 36–61 years), 33.4% of the patients were female, and the majority of implants were intermuscular (90.2%). A total of 18 patients (5.2%) developed a clinically significant pocket hematoma. There were significantly more pocket hematomas in patients with continued vitamin K antagonists (VKA) compared to patients with interrupted VKA (27.3% (6/22) vs. 4.3% (2/47), respectively, *p* = 0.01), and continuation of VKA was an independent predictor for pocket hematoma formation in the VKA group (*p* = 0.04). Moreover, continuation of dual antiplatelet therapy (DAPT) with ticagrelor was associated with significantly more pocket hematomas post-implantation compared to continuation of DAPT with clopidogrel (4/12 vs. 1/28, respectively, *p* = 0.02).

**Conclusion:**

Continuation of VKA during S-ICD implantation was associated with an increased risk of pocket hematoma formation compared to interruption of VKA. This supports the need for specific perioperative antithrombotic therapy guidelines for S-ICD implantations to reduce the risk of pocket hematomas.

**Graphical Abstract:**

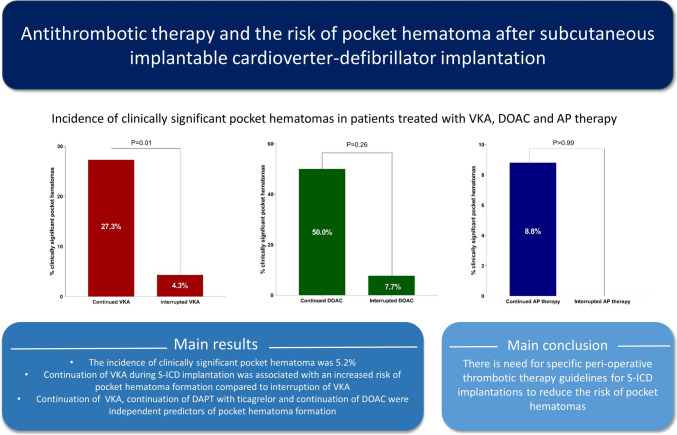

**Supplementary Information:**

The online version contains supplementary material available at 10.1007/s10840-024-01973-x.

## Introduction

Implantable cardiac defibrillator (ICD) therapy effectively prevents sudden cardiac death in patients who are at high risk of ventricular arrhythmias [1, 2]. Traditional transvenous ICDs (TV-ICD) rely on intravascular and intra-cardiac leads, which are susceptible to lead fractures and endocarditis and are associated with implant-related complications such as pneumothorax and cardiac perforation. In response to these concerns, the completely extravascular and extra-thoracic subcutaneous ICD (S-ICD) was developed [3].

The PRAETORIAN trial, which randomized S-ICD vs. TV-ICD in patients with a class I or IIa indication for ICD therapy, demonstrated that patients with S-ICDs encounter fewer lead-related complications and systemic infections [4]. However, clinically significant pocket hematomas were more frequent following S-ICD implantation. Pocket hematomas often necessitate extended hospital stays and blood transfusions and, in some cases, require surgical evacuation. Moreover, they are associated with device-related infections, in-hospital mortality, and pain often resulting in immobilization and increased hospital costs [5–7].

Many ICD recipients have comorbidities requiring anticoagulant (AC) and/or antiplatelet (AP) therapy, which increases the risk of hematoma formation during surgical procedures. Anticoagulation strategies during TV-ICD implantation have been studied extensively, advising to continue vitamin K antagonists (VKA) and continue or stop direct oral anticoagulation (DOAC) as per operator preference and/or thromboembolic risk [8–11]. Dual antiplatelet therapy should only be continued if there is an acute coronary syndrome (ACS) within 6 months or percutaneous coronary intervention (PCI) within 1 month, and the timing of the procedure should always be considered to reduce the risk of pocket hematoma [10–12].

However, little data exists regarding the optimal AC and AP strategy during S-ICD implantation to prevent pocket hematomas. Implantation of an S-ICD differs from the TV-ICD, with a larger skin incision and due to the S-ICD generator size a larger subcutaneous or intermuscular wound, consequently leading to an increased bleeding risk. This study explores the association between perioperative AC or AP management and the occurrence of pocket hematoma following S-ICD implantation in a large cohort of patients undergoing S-ICD implantation.

## Methods

### Study design and data collection

In this retrospective data analysis, all patients who underwent de novo S-ICD implantations between February 2009 and January 2023 at Amsterdam UMC were included. Patients under 18 years of age at the time of S-ICD implantation or those without follow-up data after implantation were excluded. Data regarding perioperative antithrombotic strategies, medical history, implant characteristics, occurrence of post-procedure hematoma formation, and occurrence of thromboembolic events were collected from electronic patient records. All patients attended a standard of care appointment within 2 weeks post-implantation to monitor proper wound healing. Subsequent appointments followed the standard hospital’s outpatient clinic schedule, or at increased intervals as indicated. The need for informed consent was waived by our local Medical Ethics Committee.

### Implantation

The S-ICDs were implanted under general anesthesia or monitored conscious sedation using the three- or two-incision technique [13]. All S-ICDs implanted before October 2010 had a subcutaneous position. From October 2010, the S-ICDs were implanted intermuscular, i.e., between the latissimus dorsi and serratus anterior muscles, unless a sub-serratus position was requested specifically. The day after implantation, a chest X-ray was conducted to evaluate the positioning of the generator and lead and to check for any implant-related complications. Throughout the hospital stay, the presence of pocket hematoma was carefully assessed.

### Perioperative antithrombotic protocol

The antithrombotic protocol in our tertiary center during thetime of inclusion was as follows: in patients with low thromboembolic risk, such as non-valvular atrial fibrillation (AF) and CHA2DS2VASc < 7, VKA were discontinued 3 days prior to device implantation for acenocoumarol and 5 days for fenprocoumon, and DOAC were at least withheld on the day of implantation. In patients with a high thromboembolic risk, such as AF with CHA2DS2VASc > 7, or patients with mechanical valves, antithrombotic strategies were individually assessed, often resulting in continued use of anticoagulants with a target INR of 2.0 to 3.0. In patients on antiplatelet therapy, acetylsalicylic acid was continued. P2Y12 inhibitors were by protocol advised to be interrupted several days before the procedure, if the indication for P2Y12 inhibitors was allowed. By protocol, P2Y12 inhibitors were continued when the implantation took place within 6 weeks post-ACS, percutaneous coronary intervention (PCI) with a bare metal stent, coronary artery bypass graft (CABG), percutaneous transluminal angioplasty (PTA) or cerebrovascular accident (CVA), or within 6 months post-PCI with a drug-eluting stent. However, the decision for interruption was always made in consultation with the interventional cardiologist.

### Definitions

A clinically significant pocket hematoma was defined as an accumulation of blood at the pocket site that occurs within 30 days after S-ICD implantation, necessitating actions such as applying a pressure bandage, surgical evacuation, drain insertion, change in medication or extended hospital stay, or blood transfusion [14]. Extended hospital stay was defined as either a new hospitalization or the prolongation of the stay with at least one night due to the hematoma occurrence. Baseline AC and AP therapy when administered within 7 days prior to the implant procedure and therapeutic dosing of low-molecular-weight heparin (LMWH) were included in this analysis.

### Statistical analysis

Continuous variables were tested for normality with histogram interpretation and Kolmogorov–Smirnov test. Normally distributed data is presented as means with standard deviations. Not normally distributed data is presented as median with interquartile ranges (IQR), while categorical variables are reported as frequencies with percentages. Continuous characteristics were compared between groups using the Student *t*-test for normally distributed data or the Mann–Whitney *U* test for non-normally distributed data. Binary variables were assessed using the chi-squared test or Fisher’s exact test when appropriate. Univariable logistic regression was performed to identify predictors for the occurrence of pocket hematoma following S-ICD implantation in both the total population and patients with VKA therapy pre-procedure. Covariates with *p* < 0.20 in the univariable analysis were included in the multivariable logistic regression model to test for independent predictors of pocket hematomas. Two-sided *p*-values < 0.05 were considered statistically significant. All statistical analyses were performed using IBM SPSS statistics version 28.0.1.1.

## Results

### Patient and procedure characteristics

A total of 347 patients were included of which 224 (64.6%) patients used antithrombotic therapy pre-implantation. The median age at implantation was 50 years (IQR 36–61 years), 33.4% of the patients were female, and the median BMI was 25.0 kg/m^2^ (IQR 22.5–28.3, Table 1). The vast majority of implants were intermuscular (90.2%) using the two-incision technique (80.4%), and the median procedure duration was 55 min (IQR 40–74, Table 2).

**Table 1:** Patient characteristics at baseline

	All patients	Hematoma	No hematoma	*p*-value
	*n* = 347	*n* = 18	*n* = 329	
Median age, years—(IQR)	50.0 (36.0–61.0)	56.5 (45.0–67.3)	49.0 (35.5–61.0)	0.09
Female—no. (%)	116 (33.4)	4 (22.2)	112 (34.0)	0.44
Median BMI (kg/m^2^)—(IQR)	25.0 (22.5–28.3)	24.3 (20.6–28.1)	25.1 (22.6–28.4)	0.44
eGFR < 60^a^—no. (%)	43/304 (14.1)	2/18 (11.1)	41/286 (14.3)	0.36
Smoking^b^—no. (%)	65/284 (22.9)	3/13 (23.1)	62/271 (22.9)	0.54
Diabetes mellitus—no. (%)	31 (8.9)	1 (5.6)	30 (9.1)	> 0.99
Median ejection fraction^c^—(IQR)	44.0 (27.0–57.0)	35.0 (27.0–56.5)	45.0 (27.0–57.0)	0.79
NYHA class^d^—no. (%)				0.09
I	209/300 (69.7)	8/18 (44.4)	201/282 (71.3)	
II	65/300 (21.7)	4/18 (22.2)	61/282 (21.6)	
III	18/300 (6.0)		18/282 (6.4)	
IV	2/300 (0.7)		2/282 (0.7)	
Diagnosis^e^—no. (%)				0.05
Ischemic cardiomyopathy	104 (30.0)	9 (50.0)	95 (28.9)	
Non-ischemic cardiomyopathy	63 (18.2)	1 (5.6)	62 (18.8)	
Inherited cardiac disease	110 (31.7)	2 (11.1)	108 (32.8)	
Idiopathic ventricular fibrillation	31 (8.9)	2 (11.1)	29 (8.8)	
Congenital heart disease	9 (2.6)	1 (5.6)	8 (2.4)	
Other	29 (8.4)	3 (16.7)	26 (7.9)	

*BMI* body mass index, *eGFR* estimated glomerular filtration rate, *IQR* interquartile range, *NYHA* New York Heart Association, *no.* number

^a^Missing in 43 patients

^b^Missing in 63 patients

^c^Missing in 32 patients

^d^Missing in 47 patients

^e^Missing in 1 patient

**Table 2:** Procedure characteristics and perioperative antithrombotic strategies

	All patients	Hematoma	No hematoma	*p*-value
	*n* = 347	*n* = 18	*n* = 329	
Implant type				**0.49**
Subcutaneous	24 (6.9)	1 (5.6)	23 (7.0)	
Sub-muscular	10 (2.9)	1 (5.6)	9 (2.7)	
Intermuscular	313 (90.2)	16 (88.9)	297 (90.3)	
Implant technique^a^				0.35
Three-incision	23/302 (7.6)	2/16 (12.5)	21/285 (7.4)	
Two-incision	278/302 (92.1)	14/16 (87.5)	264/285 (92.6)	
Procedure duration^b^—min. (IQR)	55 (40.00–73.8)	43.5 (38.0–52.5)	55 (40.0–75.0)	0.16
Perioperative strategy AC				
AC continued or bridged^c^	29 (8.4)	7 (38.9)	22 (6.7)	** < 0.001**
VKA continued	22 (6.3)	6 (33.3)	16 (4.9)	** < 0.001**
VKA bridged	4 (1.2)	0	4 (1.2)	> 0.99
DOAC continued	2 (0.6)	1 (5.6)	1 (0.3)	0.10
LMWH continued	1 (0.3)	0	1 (0.3)	> 0.99
AC interrupted	65 (18.7)	3 (16.7)	62 (18.8)	> 0.99
VKA interrupted	47 (13.5)	2 (11.1)	45 (13.7)	> 0.99
DOAC interrupted	13 (3.7)	1 (5.6)	12 (3.6)	0.51
LMWH interrupted	6 (1.7)	1 (5.6)	5 (1.5)	0.28
Perioperative strategy AP				
AP continued^d^	125 (36.0)	11 (61.1)	114 (34.7)	**0.02**
DAPT continued	42 (12.1)	5 (27.8)	37 (11.2)	**0.04**
DAPT continued with Ticagrelor	12 (3.5)	4 (22.2)	8 (2.4)	**0.002**
DAPT continued with Clopidogrel	28 (8.1)	1 (5.6)	27 (8.2)	> 0.99
DAPT continued with Prasugrel	2 (0.6)	0	2 (0.6)	> 0.99
ASA continued	72 (20.7)	4 (22.2)	68 (20.7)	0.77
Clopidogrel continued	38 (11.0)	3 (16.7)	35 (10.6)	0.43
Ticagrelor continued	13 (3.7)	4 (22.2)	9 (2.7)	**0.003**
Prasugrel continued	2 (0.6)	0	2 (0.6)	> 0.99
Combination of AC and AP continued	5 (1.4)	2 (11.1)	3 (0.9)	**0.02**
AP interrupted	5 (1.4)	0	5 (1.5)	> 0.99

*AC* anticoagulant, *AP* antiplatelet*, IQR* inter quartile range

^a^Missing in 46 patients

^b^Missing in 188 patients

^c^At least one anticoagulant was continued in these patients. Two patients stopped therapeutic heparin before the procedure but continued a VKA or DOAC, 4 patients were bridged

^d^At least one antiplatelet agent was continued in these patients. In five patients, the ticagrelor or clopidogrel was interrupted and ASA was continued during the procedure

### Hematomas

Eighteen (5.2%) patients developed a clinically significant pocket hematoma. The majority of patients in the hematoma group were diagnosed with an ischemic cardiomyopathy while in the no hematoma group, the majority of patients were diagnosed with an inherited cardiac disease (50% vs. 32.8% respectively, *p* = 0.05, Table 1). Eight patients had an increased bleeding risk due to other comorbidities of which four had a known malignancy and four patients were on dialysis. None of these eight patients developed a pocket hematoma.

### Anticoagulation therapy and perioperative strategy

A total of 94 (27%) patients used AC therapy prior to the implantation (Table 3). A total of 73 (21%) patients used a VKA, 15 patients (15.9%) used a DOAC, and 7 (7.4%) patients used therapeutic LMWH. One patient used a combination of therapeutic LMWH and VKA. Of the patients treated with a VKA, most patients used acenocoumarol (Table 3). The indications for VKA usage are shown in Supplementary Table 1. In 22 patients, the VKA was continued, in 47 patients, the VKA was interrupted, and in 4 patients, VKA was bridged during the procedure with high-dose LMWH (*N* = 3) or unfractionated heparin (*N* = 1) because of high thromboembolic risk.

**Table 3:** Preoperative anticoagulation and antiplatelet medication

	All patients	Hematoma	No hematoma	*p*-value
	*n* = 347	*n* = 18	*n = 329*	
Anticoagulation	94 (27.1)	10 (55.6)	84 (25.5)	**0.01**
Vitamin K antagonists	73 (21.0)	8 (44.4)	65 (19.8)	**0.02**
Acenocoumarol	67 (19.3)	7 (38.9)	60 (18.2)	0.06
Fenprocoumon	6 (1.7)	1 (5.6)	5 (1.5)	0.28
Preoperative INR^†^—(IQR)	1.4 (1.18–2.00)	2.1 (1.43–2.49)	1.3 (1.14–1.86)	**0.03**
DOAC	15 (4.3)	2 (11.1)	13 (3.95)	0.15
Dabigatran	2 (0.6)		2 (0.6)	> 0.99
Rivaroxaban	9 (2.6)	1 (5.6)	8 (2.4)	0.38
Apixaban	1 (0.3)		1 (0.3)	> 0.99
Edoxaban	3 (0.9)	1 (5.6)	2 (0.6)	0.15
LMWH (therapeutic)	7 (2.0)	1 (5.6)	6 (1.8)	0.31
Dual anticoagulation therapy^‡^	1 (0.3)	1 (5.6)	0	0.05
Antiplatelet therapy	130 (37.5)	11 (61.1)	119 (36.2)	**0.03**
Acetylsalicylic acid	118 (34.0)	9 (50.0)	109 (33.1)	0.14
Clopidogrel	41 (11.8)	3 (16.7)	38 (11.6)	0.46
Prasugrel	2 (0.6)		2 (0.6)	> 0.99
Ticagrelor	18 (5.2)	5 (27.8)	13 (4.0)	**0.001**
DAPT^§^	49 (14.1)	6 (33.3)	43 (13.1)	0.33
Combination of AC and AP use	25 (7.2)	4 (22.2)	21 (6.4)	**0.03**
AC and single AP	19 (5.5)	3 (16.7)	16 (4.9)	0.07
AC and DAPT	6 (1.7)	1 (5.6)	5 (1.5)	0.28

*AC* anticoagulant, *AP* antiplatelet, *DAPT* dual antiplatelet therapy, *DOAC* direct oral anticoagulants, *IQR* interquartile range, *LMWH* low-molecular-weight heparin

^†^INR was determined only for patients taking vitamin K antagonists (VKA). Missing in 19 patients

^‡^One patient used LMWH next to acenocoumarol

^§^Thirty-one patients used acetylsalicylic acid and clopidogrel, 16 patients used acetylsalicyclic acid and ticagrelor, and two patients used acetylsalicylic acid and prasugrel

**Fig. 1:**
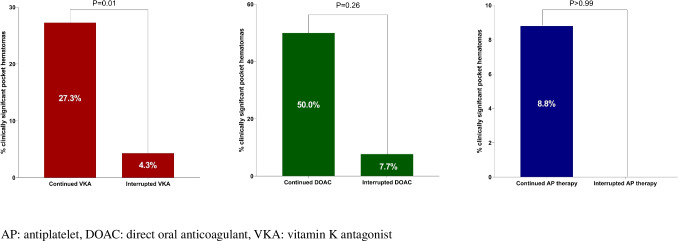
Incidence of clinically significant pocket hematomas in patients treated with VKA, DOAC, and AP therapy

The mean duration that VKA was interrupted before the procedure was 3 ± 1.5 days. None of the patients with interrupted VKA experienced a thromboembolic event post-procedure. The INR at the time of implantation in patients with continued VKA was significantly higher compared to patients with interrupted VKA (2.0 (1.3–2.7) vs. 1.3 (1.1–1.5), respectively, *p* < 0.001). There were significantly more pocket hematomas in patients with continued VKA compared to patients with interrupted VKA (Fig. 1, 27.3% (6/22) vs. 4.3% (2/47), respectively, *p* = 0.01). In patients using VKA therapy at baseline, the INR was significantly higher in the group developing a pocket hematoma compared to the group who did not (2.1 (1.4–2.5) and 1.2 (0.0–1.5), respectively, *p* < 0.001). A total of 3 pocket hematomas occurred in patients with an INR between 1 and 2, and 5 pocket hematomas occurred in patients with an INR ≥ 2.0. None of the 4 patients that were bridged with LMWH or heparin developed a pocket hematoma after S-ICD implantation.

A total of 15 (4.3%) patients used a DOAC of which most patients used rivaroxaban (Table 3). The indications for DOAC are shown in Supplementary Table 1. Two patients continued DOAC during the implantation; in the other 13 patients, DOAC was interrupted pre-procedure. The mean duration DOAC was interrupted before the implantation was 2.2 ± 1 days. None of the patients with interrupted DOAC experienced a thromboembolic event post-implantation. In patients with continued DOAC, a pocket hematoma occurred in 50% (1/2) vs. 7.7% (1/13) in patients with interrupted DOAC (*p* = 0.26, Fig. 1).

### Antiplatelet therapy and perioperative strategy

**Fig. 2:**
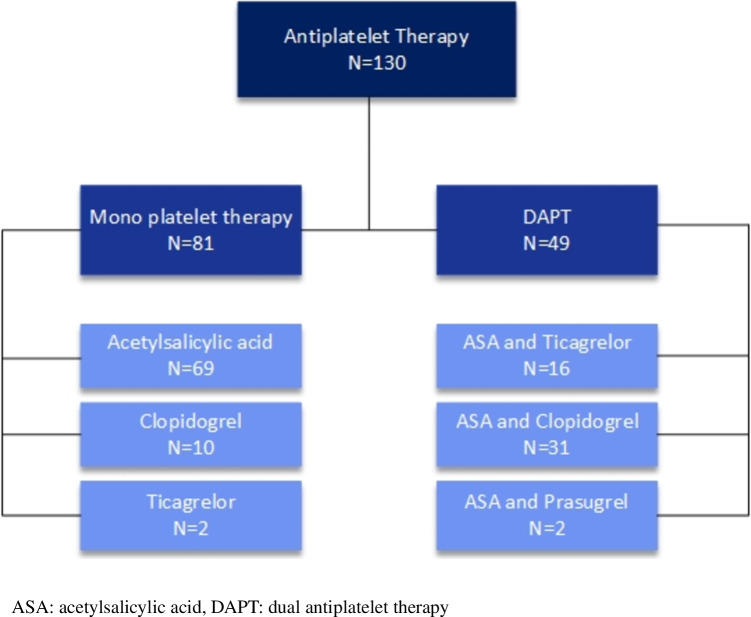
Overview of the use of antiplatelet therapy

A total of 130 (37.4%) patients used AP therapy at baseline of which 49 (14.1%) patients were on dual AP therapy (DAPT). The indications for AP usage are shown in Supplementary Table 1. The most commonly used AP was acetylsalicylic acid (90.8%) followed by clopidogrel (31.5%) (Table 3). The P2Y12 inhibitors were mainly given as part of DAPT in combination with acetylsalicylic acid (Fig. 2).

In five patients, AP therapy was interrupted pre-implantation in all other patients AP therapy, of whom 42 on DAPT was continued during the procedure. The number of patients on ticagrelor pre-procedure was significantly higher in the group of patients who developed a pocket hematoma (5/18 vs. 13/329, *p* < 0.001, Table 3). In patients with continued AP therapy, a hematoma developed in 8.8% (11/125) compared to no hematoma (0/5) in patients with interrupted AP therapy (*p* > 0.99, Fig. 1). In patients with continued DAPT therapy, a hematoma developed in 11.9% (5/42) vs. 14.3% (1/7) in patients with interrupted DAPT (*p* = 0.86). In five out of the seven patients with interrupted DAPT, ASA was continued during the implantation. Continuation of DAPT with ticagrelor was associated with significantly more pocket hematomas compared to continuation of DAPT with clopidogrel (4/12 vs. 1/28, respectively, *p* = 0.02).

### Predictors for pocket hematoma

Continuation of VKA, continuation of DOAC, and continuation of DAPT with ticagrelor were independent predictors for pocket hematoma formation in the total study population (OR 12.18 (2.95–50.26), *p* < 0.001; OR 42.54 (2.19–825.67), *p* = 0.01 and OR 18.56 (4.30–80.11), *p* < 0.001, respectively, Table 4). In patients with VKA therapy, pre-procedure continuation of VKA was an independent predictor for pocket hematoma formation (OR 6.43 (1.06–38.90), *p* = 0.04, Table 5).

**Table 4:** Independent predictors for pocket hematoma formation in the total population (*N* = 347)

	OR (95% CI)	*p*-value
Age at implantation	1.03 (0.99–1.08)	0.17
VKA continued	**12.18 (2.95–50.26)**	** < 0.001**
DOAC continued	**42.54 (2.19–825.67)**	**0.01**
LMWH continued	37.23 (0.87–1586.76)	0.06
DAPT continued ticagrelor	**18.56 (4.30–80.11)**	** < 0.001**
Clopidogrel continued*	3.02 (0.30–29.87)	0.35
Combination of AC and AP continued	1.90 (0.14–25.84)	0.63

*AC* anticoagulant, *AP* antiplatelet, *DOAC* direct oral anticoagulant, *DAPT* dual antiplatelet therapy, *LMWH* low-molecular-weight heparin, *VKA* vitamin K antagonist

^*^Patients with a single P2Y12 inhibitor continued and not as part of DAPT

**Table 5:** Independent predictors for pocket hematoma formation in patients with VKA treatment (*N* = 69)*

	OR (95% CI)	*p*-value
VKA continued	**6.43 (1.06–38.90)**	**0.04**
Combination of AC and AP continued	3.50 (0.37–33.31)	0.28

*AC* anticoagulant, *AP* antiplatelet, *VKA* vitamin K antagonist

^*^Patients with bridging were excluded

### Pocket hematoma–related interventions and complications

Among the 18 patients with a pocket hematoma, 1 patient needed a surgical evacuation, 1 patient needed a blood transfusion, and 2 patients had a change in medication. Sixteen patients received a pressure bandage. In 7 patients (2.0%), the pocket hematoma resulted in prolonged or new hospitalization. A pocket hematoma resulted in a pocket infection in one patient with increased infection parameters. In 50% of the patients with hematoma, the pocket was documented as very painful. All these patients required additional pain medication, and two patients experienced a vasovagal reaction due to the pain. In 5 hematoma patients, the hemoglobin levels were reported of which 3 patients showed a decrease in hemoglobin of more than 1 mmol/l. One patient experienced a failed defibrillation test post-implantation which resulted in a resuscitation setting. The sensing issues during DFT were related to the accumulation of blood in the pocket. After resolution of the hematoma, the DFT was successfully repeated.

## Discussion

This study showed that 5.2% of patients developed a pocket hematoma after subcutaneous ICD implantation. Continuation of VKA was associated with an increased risk of pocket hematoma formation during S-ICD implantation compared to interruption of VKA. Continuation of VKA, continuation of DAPT with ticagrelor, and continuation of DOAC were independent predictors for hematoma formation in the total population. Furthermore, continuing DAPT with ticagrelor was associated with significantly more pocket hematomas compared to continuation of DAPT with clopidogrel.

The rate of pocket hematomas was higher compared to a previous large S-ICD registry and the two major RCT trials, which showed rates between 0.8 and 2.1% [4, 15, 16]. Potential reasons include variations in definitions for clinically significant pocket hematomas, variations in perioperative antithrombotic protocols, differences in patient selection, and diverse positioning techniques of the S-ICD with the majority of S-ICDs implanted intermuscular in our study.

### Anticoagulation therapy

In this study, continuation of VKA was associated with an increased risk of pocket hematoma formation (27.3% vs. 4.3%). Two studies on anticoagulation strategies in S-ICD patients also reported significantly more pocket hematomas in patients with continued warfarin (25% vs. 1.8% and 26% vs. 4%, respectively), and in one study, continuation of warfarin therapy was a predictor for pocket hematoma formation (OR 11.1 (1.7–74.3), *p* = 0.01) [17–19]. In contrast, our study utilized acenocoumarol or fenprocoumon, reflecting global variations in VKA preferences [20]. While warfarin offers a longer half-life time and more stable INR levels, our study showed comparable pocket hematoma rates in patients on continued acenocoumarol or fenprocoumon during S-ICD implantation with a predominantly intermuscular implant position [21, 22].

The increased risk of pocket hematoma formation with continuation of VKA vs. interrupted contrasts findings from TV-ICD trials. In transvenous devices, rates of pocket hematoma in patients with VKA continued to range from 0 to 5%, and pocket hematoma rates in patients with interrupted VKA range from 0 to 3% showing no significant differences and overall lower event rates of pocket hematoma compared to S-ICD patients [23–27]. This difference may be attributed to procedural and position disparities between the S-ICD and TV-ICD implantation. While the TV-ICD is subcutaneously positioned, the S-ICD is mainly placed intermuscular, between muscular layers on the thorax’s lateral side, where there might be a more dense vascular network. Another difference between the TV-ICD and S-ICD is the larger generator of the S-ICD which requires a bigger incision and pocket. Limited visibility in a deeper lateral pocket could also lead to easier oversight of bleeding. Additionally, applying a post-implantation preventive pressure bandage for an S-ICD is more challenging than for a TV-ICD and therefore less often applied preventively in S-ICD patients.

There might be a higher risk of pocket hematomas due to the pharmacokinetic profile of DOAC therapy with peak plasma levels several hours after DOAC intake [28]. Unfortunately, as there were only two patients on continued DOAC therapy in our study, it is not possible to draw meaningful conclusions about the risk of pocket hematoma formation for this treatment strategy. Previous transvenous device studies on DOAC therapy and pocket hematoma formation are inconclusive. One recent study reported that continuation of DOAC is associated with a higher percentage of clinically significant hematoma compared to interruption of DOAC albeit not statistically significant (4.5% vs. 1.7%, *p* = 0.06) [29]. On the other hand, the BRUISE-CONTROL-2 trial showed no difference in the occurrence of pocket hematomas in transvenous device patients with continued vs. interrupted DOAC therapy [9]. However, more studies in both S-ICD and TV-ICD patients are needed to give better insight on the preferred DOAC strategy to reduce the risk of pocket hematoma formation.

Currently, the HRS/ESC guidelines do not differentiate between S-ICD and TV-ICD, and the advice to continue AC, DOAC, or VKA, during device implantation, is based on TV-ICD trials [10]. Our findings highlight the necessity for distinct trials and guidelines for S-ICD implantation. We recommend, until further studies are performed, to withholding VKA therapy in S-ICD patients with a low thromboembolic risk to reduce the risk of pocket hematoma formation after implantation. Interrupting anticoagulation is not recommended in patients with a high thromboembolic risk, as thromboembolic events can have severe consequences. When considering interrupting anticoagulation, it is crucial to always carefully assess the patient’s individual thromboembolic risk*.* We would also recommend avoiding bridging of VKA; while our numbers were too small to find an association with pocket hematoma formation in S-ICD patients, earlier studies in TV-ICD patients did show a very strong association between bridging and the occurrence of pocket hematoma formation [8].

### Antiplatelet therapy

In the study of Sheldon et al., clopidogrel emerged as an independent predictor for pocket hematoma formation after S-ICD implantation while in our study continuation of DAPT with specifically ticagrelor was predictive for pocket hematoma formation and showed more pocket hematomas compared to continuation of DAPT with clopidogrel [18]. This difference might be explained due to the low number of patients on ticagrelor in the study of Sheldon et al. Given that ticagrelor is a more potent P2Y12 inhibitor than clopidogrel, it is expected that the risk of bleeding would be higher with ticagrelor, especially in combination with acetylsalicylic acid. Our results suggest that switching from ticagrelor to clopidogrel as part of DAPT before S-ICD implantation might reduce the risk of pocket hematoma. This could be particularly relevant for secondary prevention patients, in which postponing implantation is often undesirable and interrupting DAPT therapy is frequently contra-indicated due to recent ACS or PCI.

#### Predictors and complications

Continuation of VKA, DOAC, and DAPT with ticagrelor were independent predictors for pocket hematoma formation. Interestingly, an intermuscular or submuscular position of the S-ICD was not associated with hematoma formation. This finding is of interest, as an intermuscular position might be expected to result in more hematomas than a subcutaneous position, due to the position between the vascularized muscles. These results support the use of the intermuscular implantation technique, as it is also associated with fewer complications and inappropriate shocks [30].

In conclusion, it is clinically relevant and in patients’ best interest to try and reduce the risk of pocket hematoma formation after S-ICD implantation. Our study showed the negative effects pocket hematomas can have on patients’ health, including re-intervention, blood transfusion, and infection risk as well as extra need for pain medication and risk of DFT failure resulting in resuscitation.

## Limitations

Due to the retrospective nature of this study, the data obtained from electronic patient records might introduce potential biases due to missing information. The reason for interruption or continuation of AC during implantation was not randomized or protocolized, and therefore, unknown clinical factors might have contributed to the choice of anticoagulation strategy and the risk of pocket hematoma formation. Moreover, we describe a relatively young ICD patient population without major comorbidities of which a relatively large proportion had been diagnosed with inherited cardiac disease. Due to the small numbers of patients with bridging of AC or with continued DOAC and interrupted AP therapy, no regression analysis could be performed in these groups specifically, and no conclusion on strategy can be made from the data in this study. Studies with larger cohorts, particularly including more patients on DOAC therapy, could provide valuable insights for future anticoagulation guidelines for S-ICD patients. The antithrombotic protocol used in our center has recently been adapted due to new insights.

## Conclusion

In S-ICD patients, the incidence of clinically significant pocket hematoma was 5.2%. Continuation of VKA during S-ICD implantation was associated with an increased risk of pocket hematoma formation compared to interruption of VKA. Continuation of VKA, continuation of DOAC, and continuation of DAPT with ticagrelor were independent predictors of pocket hematoma formation. Multiple negative effects on patient health including re-intervention, infection, pain, and failed DFT were reported after pocket hematoma formation. These findings support the need for specific perioperative thrombotic therapy guidelines for S-ICD implantations to reduce the risk of pocket hematomas.

## Supplementary Information

Below is the link to the electronic supplementary material.

Supplementary file1 (DOCX 279 KB)

## Data Availability

The data underlying this article will be shared on reasonable request to the corresponding author.

## Declarations

Data was collected retrospectively and the need for informed consent was waived by our local Medical Ethics Committee.
