# Supplementary material for: Antithrombotic therapy and the risk of pocket hematoma after subcutaneous implantable cardioverter-defibrillator implantation
Source: J Interv Card Electrophysiol. 2025 Jan 16;68(7):1437–46. doi: 10.1007/s10840-024-01973-x (PMC12436547; doi:10.1007/s10840-024-01973-x)
Supplement: Supplementary file 1 — Supplementary file1 (DOCX 279 KB) [file 10840_2024_1973_MOESM1_ESM.docx]

Supplementary material

**Supplementary Figure 1. Overview of antithrombotic therapy and strategy in patients with pocket hematomas 2**

**Supplementary Table 1. Indications for anticoagulation and antiplatelet use pre-procedure** **3**

**Supplementary Table 2. Univariable predictors for pocket hematoma in the total population 4**

**Supplementary Table 3. Univariable predictors for pocket hematoma in patients with VKA treatment 4**

Supplementary Figure 1. Overview of antithrombotic therapy and strategy in patients with pocket hematomas

**
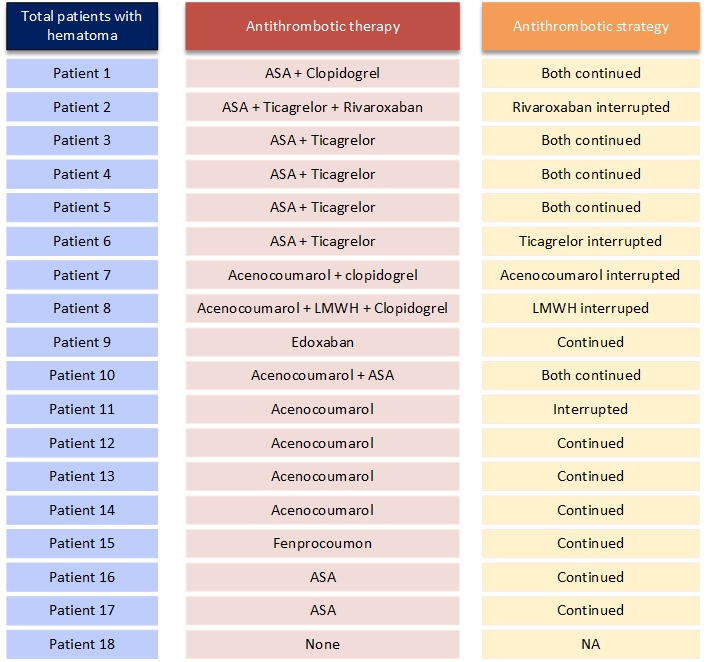
**

Supplementary table 1. Indications for anticoagulation and antiplatelet use pre-procedure

|  | **All patients** | **Hematoma** | **No hematoma** |
| --- | --- | --- | --- |
|  | **n = 347** | **n = 18** | **n = 329** |
| Indications for VKA | N = 73 | N=8 | N=65 |
| Intra cardiac thrombus | 28 (38.4) | 3 (37.5) | 25 (38.5) |
| Atrial fibrillation | 23 (31.5) | 2 (25.0) | 21 (32.3) |
| Left ventricular aneurysm | 14 (19.2) | 2 (25.0) | 12 (18.5) |
| Prosthetic heart valve | 6 (8.2) | 2 (25.0) | 4 (6.2) |
| TIA/CVA | 4 (5.5) | 2 (25.0) | 2 (3.1) |
| Pulmonary Embolism or DVT | 4 (5.5) | 0 | 4 (6.2) |
| Other^†^ | 6 (8.2) | 0 | 6 (9.2) |
| Indications for DOAC | N = 15 | N=2 | N=13 |
| Intra cardiac thrombus | 2 (13.3) | 0 | 2 (15.4) |
| Atrial fibrillation | 10 (66.7) | 0 | 10 (76.9) |
| Left ventricular aneurysm | 1 (6.7) | 0 | 1 (7.7) |
| Pulmonary embolism | 4 (26.7) | 3 | 1 (7.7) |
| Other^‡^ | 1 (6.7) | 0 | 1 (7.7) |
| Indication for LMWH | N = 7 | N=1 | N=6 |
| Atrial fibrillation | 3 (42.9) | 1 (100) | 2 (33.3) |
| Prosthetic heart valve | 2 (28.6) | 0 | 2 (33.3) |
| Other^§^ | 2 (28.6) | 0 | 2 (33.3) |
| Indications for AP | N = 130 | N=11 | N=119 |
| Primary prevention CVD | 3 (4.1) | 0 | 3 (4.6) |
| Coronary artery disease | 107 | 10 (55.6)^#^ | 97 (29.5)^¥^ |
| TIA/CVA | 9 (2.6) | 1 (5.6) | 8 (2.4) |
| Other^¶^ | 11 (15.1) | 0 | 11 (16.9) |

AP: antiplatelet, CVA: cerebrovascular accident, CVD: coronary vascular disease, DOAC: direct oral anticoagulants, DVT: deep vein thrombosis, IQR: inter quartile range, LMWH: low molecular weight heparin, TIA: transient ischemic attack, VKA: vitamin K antagonist. † In 5 patients due to reduced LVEF and in 1 patient due to phospholipid syndrome, ‡ One patient had a thrombophlebitis with multiple infected thrombi, § one patient used LMWH because of a recent EFO and one patient because a ventricular aneurysm, ¶ One patient post cardiac surgery (ALCAPA), 1 patient due to a biobental prosthesis , 2 patients post ablation, 1 patient with coronary spasm, # 5 with ACS en 5 post PCI, ¥ 59 ACS, 2 stable angina pectoris, 27 post PCI, 9 post CABG.

Supplementary table 2. Univariable predictors for pocket hematoma in the total population

|  | **OR (95% CI)** | **P-value** |
| --- | --- | --- |
| Age | 1.029 (0.996-1.065) | **0.09** |
| Sex | 1.806 (0.581-5.617) | 0.31 |
| BMI | 0.980 (0.877-1.095) | 0.72 |
| eGFR < 60 | 0.878 (0.195-3.959) | 0.87 |
| Smoking | 1.314 (0.748-2.309) | 0.34 |
| Implantation type subcutaneous vs intermuscular and sub-muscular | 1.278 (0.163-10.034) | 0.82 |
| VKA continued | 9.781 (3.252 – 29.421) | **<0.001** |
| VKA bridged | NA* | 0.99 |
| DOAC continued | 19.294 (1.157-321.873) | **0.04** |
| LMWH continued | 19.294 (1.157-321.873) | **0.04** |
| DAPT continued | 3.035 (1.024-8.998) | **0.05** |
| DAPT continued with Ticagrelor | 11.464 (3.081-42.664) | **<0.001** |
| DAPT continued with Clopidogrel | 0.658 (0.084-5.136) | 0.69 |
| DAPT continued with Prasugrel | NA* | 0.99 |
| ASA continued | 1.097 (0.350-3.439) | 0.87 |
| Clopidogrel continued | 5.016 (0.984-25.568) | **0.05** |
| Ticagrelor continued | NA* | >0.99 |
| Combination of AC and AP continued | 13.583 (2.118-87.103) | **0.01** |

* The 95% Confidence Interval is not provided for p-values close to 1.

Supplementary table 3. Univariable predictors for pocket hematoma in patients with VKA treatment

|  | **OR (95% CI)** | **P-value** |
| --- | --- | --- |
| Age | 0.983 (0.933 – 1.035) | 0.51 |
| Sex | 1.159 (0.213 – 6.315) | 0.86 |
| BMI | 0.915 (0.761 – 1.101) | 0.35 |
| eGFR < 60 | 0.480 (0.054 – 4.237) | 0.51 |
| Smoking | 1.410 (0.563 – 3.531) | 0.46 |
| Implantation type subcutaneous vs intermuscular and sub-muscular | 0.237 (0.019-2.964) | 0.26 |
| VKA continued | 8.437 (1.543 – 46.138) | **0.01** |
| Combination of AC and AP continued | 9.833 (1.166 – 82.939) | **0.04** |
